# Supplementary material for: Learning curve and surgical outcome of robotic assisted colorectal surgery with ERAS program
Source: Sci Rep. 2022 Nov 29;12:20566. doi: 10.1038/s41598-022-24665-w (PMC9709162; doi:10.1038/s41598-022-24665-w)
Supplement: Supplementary file 2 — Supplementary Information 2. [file 41598_2022_24665_MOESM2_ESM.zip › RAL Raw data-Ñ[▒K/053/Patho_ñw╝╨░O▒Kñσ.pdf]

[redacted]

Pathologic diagnosis:
1. Rectum and anus, robotic APR ---- Status post CCRT with residual malignant melanoma of rectum. Tumor invades to muscular layer, close to anorectal junction, and metastasized to perirectal lymph nodes (4/15). The resection margins are free of tumor.
2. IMA root, dissection --- Unremarkable fibrovasculoadipose tissue.
3. "Peritoneium", resection --- Endometriosis.
4. Lymph node, para-aortic, dissection --- Unremarkable (0/1).

Ancillary study for diagnosis:
IHC stains for MART-1 (+ on melanocytes) and SOX10 (+ on melanocytes) done on section G.

Prognostic and predictive factor:
1. Tumor size: 5x4.5 cm.
2. Depth of invasion: Muscularis propria.
3. Histologic grade: Not applicable.
4. Angiolymphatic invasion: Not identified.
5. Perineural invasion: Not identified.
6. Tumor deposits (discontinuous extramural extension): Not identified.
7. Macroscopic tumor perforation: Not identified.
8. Surgical Margins: a) Proximal Margin: Uninvolved by invasive carcinoma, distance of tumor from margin: 10 cm. b) Distal Margin: Uninvolved by invasive carcinoma, distance of tumor from margin: 3 cm. c) Radial or Mesenteric Margin: Uninvolved by invasive carcinoma, distance of tumor from margin: 0.6 cm.
9. Lymph node status: Involved (4/16).
10. Extranodal involvement: Absent.
11. Tumor regression grading S/P CCRT: Residual cancer with evident tumor regression but more than single cells or rare small groups of cancer cells (partial response, score 2).
12. Type of polyp in which invasive carcinoma arose: Not applicable.
13. Additional pathologic findings: Not identified.
14. Pathological TNM Stage: Not applicable.
15. TNM descriptors: Not applicable.

Gross description:
The specimen consists of 1) a segment of rectum, and a piece of anal soft tissue. The rectum with perirectal soft tissue, measured 15 cm in length and 5 cm in proximal cut end circumference, 7.5 cm in distal circumference. The anal soft tissue measured 7x3.5x3.5 cm. A gray tan firm annular elevated and centrally ulcerative tumor, 5x4.5 cm, is noted, 3 cm to the nearest cut end, and close to anorectal junction. On cross section, the tumor invades to the muscular layer with 6 mm to the radial margin. The rectum cut end and anal skin are free of tumor grossly. 2) IMA root, a piece of tan yellow soft tissue, 1x0.5x0.3 cm. 3) "peritoneum", a piece of tan soft tissue, 3.5x1.5x1 cm. 4) "para-aortic LN", a piece of tan soft tissue, 0.5x0.5x0.3 cm. Representative part for section: A) specimen 2 all for section B) specimen 3 C) specimen 4 all for section D) rectum cut end E) anal skin cut end F+G+H) tumor with anorectal junction J-K) tumor and CFM L-N) tumor P-Q) lymph nodes.

Note:
This case has been peer reviewed by two doctors.
#T-59300_2 #M-87203_2 9 1105 M0000F
#T-C4560_2 #M-87203_2 9 1105 000000

已電子簽章

敬告：本單僅供醫師參考使用，不具任何申請效力！！
請病患妥為保管，若不慎遺失需自負個人資料外洩責任！
